# Supplementary material for: Content-rich biological network constructed by mining PubMed abstracts
Source: BMC Bioinformatics. 2004 Oct 8;5:147. doi: 10.1186/1471-2105-5-147 (PMC528731; doi:10.1186/1471-2105-5-147)
Supplement: Additional File 2 — The original results of the above study (non-essential files are deleted to keep the file size under the limit set by BMC bioinformatics). [file 1471-2105-5-147-S2.bz2 › chilibotAdditionalFile2/dip20/496ID10187774E10659/html/ACVR2_ACVRL1.html]

 


 **ACVR2** and **ACVRL1** 
  
Found 62 abstracts in PubMed, retrieved 20.  
 

 What does Google say? 
 PDF only 
| .edu only 

---

**Interactive relationship** (e.g. stimulation, inhibition, etc)

**Neutral relationship**- Activin receptor  [ **ACVR2** ]  like kinase 1 ALK 1  [ **ACVRL1** ] , the gene mutated in HHT  [ **ACVRL1** ]  type 2 HHT2  [ **ACVRL1** ] , is a serine threonine kinase receptor type I of the TGF beta superfamily, specifically expressed on endothelial cells.  Ref: 12700602 Eur J Hum Genet, 2003
- HHT  [ **ACVRL1** ]  type 2 is caused by loss of function mutations in activin receptor  [ **ACVR2** ]  like kinase 1  **ACVRL1**  or ALK1  [ **ACVRL1** ] .  Ref: 12588795 Hum Mol Genet, 2003
- Hereditary hemorrhagic telangiectasia HHT  [ **ACVRL1** ]  is a vascular disorder in humans which has been mapped to two genes, endoglin and activin receptor  [ **ACVR2** ]  like kinase 1 ALK 1  [ **ACVRL1** ]  both of which mediate signaling by transforming growth factor beta ligands in vascular endothelial cells.  Ref: 12667943 Cardiovasc Res, 2003

**Non-interactive relationship** (e.g. studied together, co-existance, homology, etc.)

- We show that vbg encodes activin receptor  [ **ACVR2** ]  like kinase 1  **Acvrl1** .  Ref: 12050147 Development, 2002
- In the two largest families, we used microsatellite markers to test for linkage to genes encoding TGF beta receptor proteins, including endoglin and activin receptor  [ **ACVR2** ]  like kinase 1 ALK1  [ **ACVRL1** ] , and BMPR2.  Ref: 11484689 N Engl J Med, 2001
- Recent studies have identified mutations of the bone morphogenetic protein receptor 2 BMPR2 gene and the activin receptor  [ **ACVR2** ]  like kinase 1 ALK1  [ **ACVRL1** ]  gene, that affect the transforming growth factor beta TGF beta receptor superfamily, a group of transmembrane signaling molecules with serine threonine kinase activity that are involved in the regulation of cell growth.  Ref: 12624796 Z Rheumatol, 2003
- A mechanistic explanation for increased pSmad1 5 staining during inflammation was provided by the upregulated expression of all the BMP type I receptors, i.e., activin receptor  [ **ACVR2** ]  like kinase ALK  [ **ACVRL1** ]  2, ALK3, and ALK6, in the inflamed bronchial epithelial cells.  Ref: 12151307 Am J Respir Cell Mol Biol, 2002
- This was accompanied by strong upregulation of the activin receptor  [ **ACVR2** ]  ALK  [ **ACVRL1** ]  4 ActR IB and redistribution of the TGF beta responsive ALK  [ **ACVRL1** ]  5 TbetaR I.  Ref: 11472976 Am J Respir Cell Mol Biol, 2001
- Two related genes, endoglin and activin receptor  [ **ACVR2** ]  like kinase ALK 1  [ **ACVRL1** ] , have been mapped to chromosomes 9q34 and 12q13, respectively.  Ref: 11802521 J Formos Med Assoc, 2001
- Activin receptor  [ **ACVR2** ]  like kinase ALK 1  [ **ACVRL1** ] , ALK  [ **ACVRL1** ]  2, ALK  [ **ACVRL1** ]  3, and ALK  [ **ACVRL1** ]  6 were able to reproduce the hypertrophic maturation of chondrocytes induced by BMP 2.  Ref: 12082094 J Biol Chem, 2002
- We expressed the kinase domain of the TGF beta type I receptor activin receptor  [ **ACVR2** ]  like kinase ALK  [ **ACVRL1** ]  5 and the substrate, Smad3, and determined that SB 431542 is a selective inhibitor of Smad3 phosphorylation with an IC50 of 94 nM.  Ref: 12065755 Mol Pharmacol, 2002
- Surprisingly, activin had no effect on follistatin mRNA levels, despite the fact that FS D1h cells express ActRII  [ **ACVR2** ] , ActRIIB, and ALK  [ **ACVRL1** ]  4 ActRIB.  Ref: 12538636 Endocrinology, 2003
- A preliminary investigation was performed to exclude association between the predisposition and certain candidate genes including KDR kinase insert domain receptor, TEK TEK tyrosine kinase endothelial,  **ACVRL1**  activin receptor  [ **ACVR2** ]  like kinase 1, ENG endoglin and FLT4 fms like tyrosine kinase 4.  Ref: 12752572 Clin Genet, 2003
- SB 431542 is a potent and specific inhibitor of transforming growth factor beta superfamily type I activin receptor  [ **ACVR2** ]  like kinase ALK  [ **ACVRL1** ]  receptors ALK4, ALK5, and ALK7.  Ref: 12065756 Mol Pharmacol, 2002
- Reverse transcription polymerase chain reaction of PASMC mRNA detected transcripts for type I TGF betaRI, Alk 1  [ **ACVRL1** ] , ActRI, and BMPRIB and type II TGF betaRII, BMPR II, ActRII  [ **ACVR2** ] , ActRIIB receptors.  Ref: 11502704 Circulation, 2001
- Most cases are caused by mutations in the endoglin gene on chromosome 9 HHT  [ **ACVRL1** ]  type 1 or the activin receptor  [ **ACVR2** ]  like kinase 1 gene on chromosome 12 HHT  [ **ACVRL1** ]  type 2, which leads to telangiectases and arteriovenous malformations AVM of the skin, mucosa, and viscera.  Ref: 11773580 Pediatrics, 2002
- Here, we characterize a small molecule inhibitor SB 431542 that was identified as an inhibitor of activin receptor  [ **ACVR2** ]  like kinase ALK  [ **ACVRL1** ]  5 the TGF beta type I receptor.  Ref: 12065756 Mol Pharmacol, 2002
- Constitutively active forms of BMP type I receptors BMPR IA and BMPR IB and those of activin receptor  [ **ACVR2** ]  like kinase ALK 1  [ **ACVRL1** ]  and ALK  [ **ACVRL1** ]  2 maintained differentiation of chondrocytes in the presence of retinoic acid.  Ref: 12565908 Biochem Biophys Res Commun, 2003
- Activin receptor  [ **ACVR2** ]  like kinase 5 ALK  [ **ACVRL1** ]  5 is a TGF beta type I receptor expressed in various TGF beta responsive cells.  Ref: 12384983 J Cell Physiol, 2002
